# Early administration of hydrocortisone replacement after advent of septic shock is a major determinant of final outcome

**DOI:** 10.1186/cc9832

**Published:** 2011-03-11

**Authors:** C Katsenos, I Tsagkaris, A Antonopoulou, A Savva, A Michaloglou, E Giamarellos-Bourboulis, A Armaganidis, K Mandragos

**Affiliations:** 1 Korgialeneio Benakeio Hospital, Athens, Greece; 2 University of Athens, Medical School, Athens, Greece; 3 Attikon University Hospital, Athens, Greece

## Introduction

The CORTICUS trial doubts the value of hydrocortisone replacement for final outcome of septic shock [1]. We hypothesized that the time of starting hydrocortisone may impact on the final outcome.

## Methods

Retrospective analysis was made of prospectively collected data for 41 patients with septic shock (ACCP/SCCM 1992 definition) in the past year in two ICUs. Hydrocortisone was infused as suggested [2]. The time lapsing from start of vasopressors until start of hydrocortisone was determined by the patients' charts.

## Results

**Figure 1:**
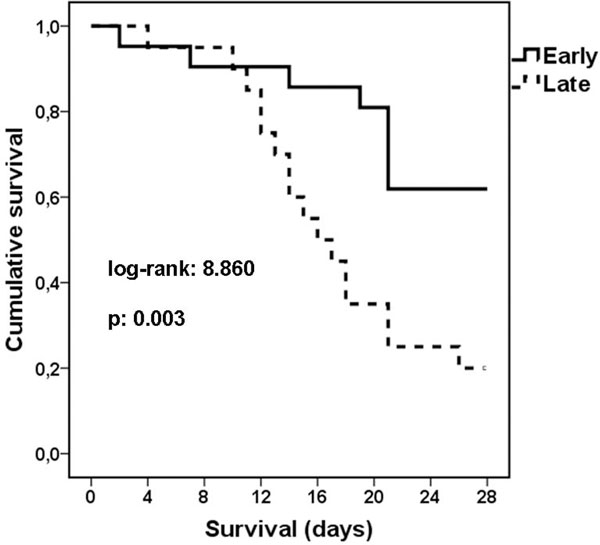
**Survival in relation to start of hydrocortisone**.

Early start of hydrocortisone was determined by the quartiles of lapsing time as less than 24 hours. The impact of early start is shown in Figure 1. The mean APACHE II score for patients in early start was 22.09 and for patients in late start was 18.33 (*P = *NS). Cox regression analysis revealed that the only factor affecting final outcome was early start of hydrocortisone (HR: 4.85, 95% CI: 1.11 to 21.22, *P *= 0.036) as opposed to appropriateness of antimicrobial treatment (HR: 2.80, 95% CI: 0.56 to 13.91, *P = *NS).

## Conclusions

Despite the observational approach, early start of hydrocortisone replacement in septic shock is a critical factor for outcome.
